# Novel unconditioned prosocial behavior in prairie voles (*Microtus ochrogaster*) as a model for empathy

**DOI:** 10.1186/s13104-018-3934-0

**Published:** 2018-12-04

**Authors:** Lucas A. Stetzik, Alana W. Sullivan, Heather B. Patisaul, Bruce S. Cushing

**Affiliations:** 1 University of Akron, 302 E. Buchtel Ave, Akron, OH 44325 USA; 2 University of Florida, P.O. Box 100267, Gainesville, FL 32610-0267 USA; 3 North Carolina State University, Raleigh, NC 27695 USA; 4 University of Texas at El Paso, 500W. University Ave, El Paso, TX 79968 USA

**Keywords:** Empathy, Prosocial, Oxytocin, Dopamine, Rodent model, Vole

## Abstract

**Objective:**

In this study, empathy is quantified using a novel social test. Empathy and prosocial behavior are linked to the expression of oxytocin in humans and rodent models. Specifically, prosocial behavior in prairie voles (*Microtus ochrogaster*) has been linked to the expression of oxytocin in the paraventricular nucleus of the hypothalamus. The animal’s behavior was considered empathic if it spends significantly more time attempting to remove a loos fitting restraint (tether) from the stimulus animal than time in contact with a, simultaneously presented, non-social object similar to the tether. The behavioral data was cross-referenced with the number of neurons expressing oxytocin and arginine vasopressin, as well as the density of dopaminergic neurons (identified by the expression of tyrosine hydroxylase), in the paraventricular nucleus of the hypothalamus. These proteins influence empathic behavior in humans, non-human primates, rats, mice, and prairie voles.

**Results:**

The consistency between neuroanatomical mechanisms linked to empathy, and the durations of time spent engaging in empathic contact, support the prediction that the empathic contact in this test is a distinct prosocial behavior, lacking prior behavioral training or the naturally occurring ethological relevance of other prosocial behaviors, and is a measure of empathy.

**Electronic supplementary material:**

The online version of this article (10.1186/s13104-018-3934-0) contains supplementary material, which is available to authorized users.

## Introduction

In humans, observing distress drives signaling between the cingulate cortex, prefrontal cortex, and limbic regions, such as the amygdala and hypothalamus [1]. This correlates with self-reported empathy, an external expression of which is prosocial behavior [2]. Prosocial behaviors have been linked to the expression of oxytocin (OT) in humans [3, 4]. In prairie voles (*Microtus ochrogaster*), OT regulates prosocial behaviors, such as partner preference formation, alloparental care, and consoling behavior [5–8]. Specifically, prosocial behavior in prairie voles has been linked to the expression of OT in the paraventricular nucleus of the hypothalamus (PVN) [9, 10]. The PVN signals to the pituitary and the supraoptic nucleus, triggering a cascade of signaling molecules that modulate the regulatory mechanisms of steroid-dependent prosocial behaviors such as pair-bonding and alloparental care, through estrogen receptor alpha (ERα) [11]. It has been hypothesized that OT’s role as an empathogen may have first evolved in the context of kinship relationships, and that the ability to accurately infer others emotions may provide an advantage through the ‘trust-effect’ of OT [12]. The mechanism by which OT influences empathy is currently unknown, but if it is an extension of the mechanism by which it regulates other steroid-dependent prosocial behaviors, it is likely to modulate corticolimbic signaling patterns [13] through ERα.

In this study, neurons expressing OT, arginine vasopressin (AVP), or tyrosine-hydroxylase (TH) (dopaminergic neurons), were counted in the PVN of prairie voles. While OT, AVP and dopamine in the PVN are linked to social behaviors like exploratory sniffing [14–16], only OT and AVP in the PVN are linked to empathy [7, 12, 17–20]. A role for dopaminergic PVN neurons, in the context of empathy, remains unexplored. Dopamine in the anterior cingulate cortex [21] and nucleus accumbens, however, is important in empathy-like responses to observational fear. In prairie voles, males have more dopaminergic neurons in the PVN than females [22], but not OT neurons [11]. Here we sought to determine if there is an unconditioned prosocial behavior, distinct from other prosocial behaviors, that is indicative of empathy and linked to PVN OT neurons.

**Fig. 1:**
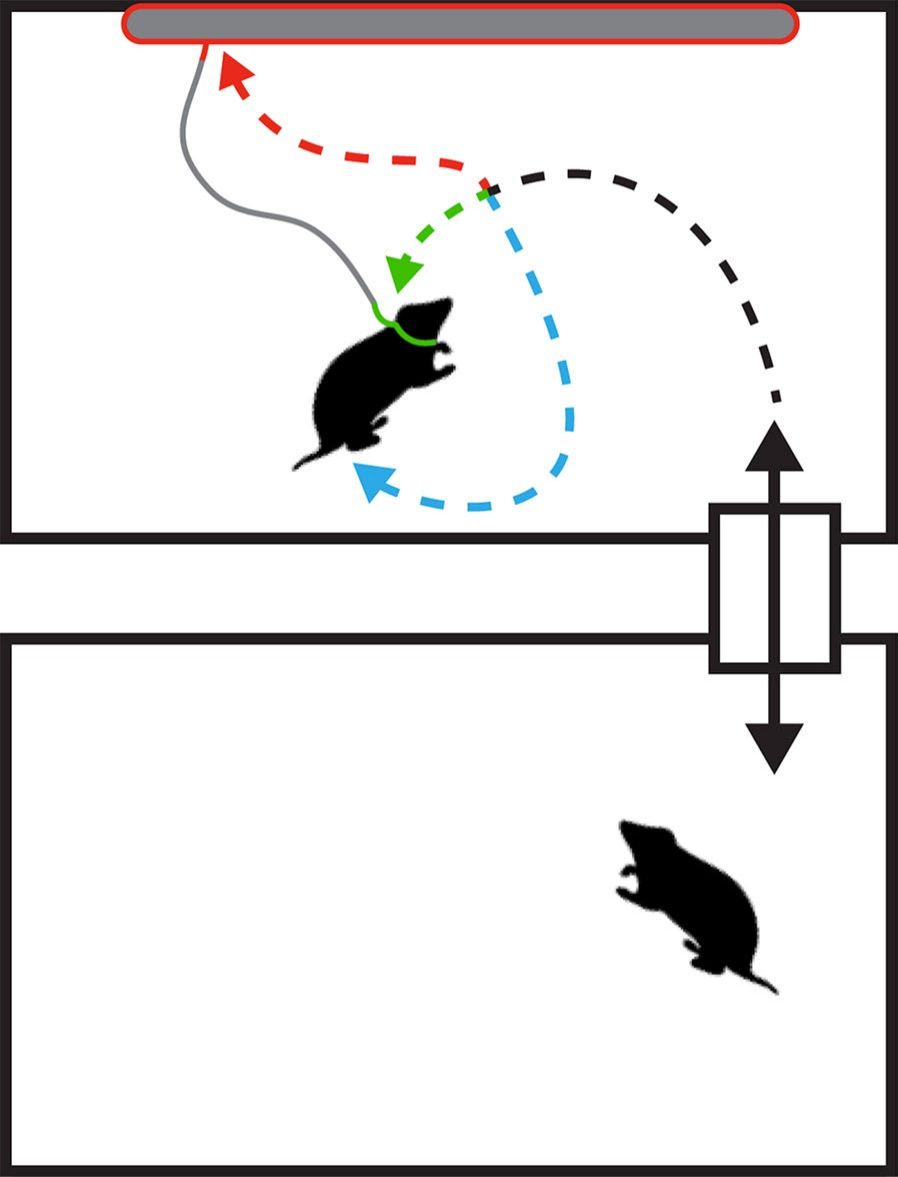
Novel social test: bottom rectangle depicts non-social cage for test animal, top rectangle depicts social cage with stimulus animal tethered inside, and small rectangle with vertical double arrow depicts connecting tube. Blue arrow indicates exploratory contact, green arrow indicates empathic contact, and red arrow indicates contact with non-social object. Elongated grey oval with red outline indicates non-social object. Portion of animal tether/collar colored green indicates object of empathic consequence

Quantifying empathic behavior in rodents often requires specific training reinforced by reward [2, 7, 23]. In these studies, a test animal observes a stimulus animal expressing distress from foot shock [20, 23] or forced restraint [2], and the test animal can perform a specific task to eliminate the distress, such as pulling a lever. The training and reward confounds empathic behavior as, potentially, a product of training. Other rodent models of empathy utilize a freezing response in an observer animal that witnesses distress [7, 8, 20, 24]. However, freezing behavior can result from social transmission of fear [25, 26] or empathy. Allogrooming between a distressed animal and cage mate, or conspecific is the strongest prosocial measure of empathy [7, 27], but grooming can be aggressive and dominance driven [28]. The functions of grooming complicate its interpretation in rodent models of dominance driven social hierarchy, such as rats [26]. The advantage of the behavior used in the present study is that it does not require training or social fear, and lacks known alternative ethological functions. Here, we use a novel social test similar to those used in social preference tests [29, 30] (Fig. 1). The test animal is given no specific training or reward, and the behavior considered empathic is time spent attempting to remove a loose fitting restraint tether from an unrelated, same sex, size and age matched stimulus animal, vetted as non-aggressive.

To establish this behavior as empathic, time spent engaging in exploratory sniffing and time spent in contact with a non-social object were quantified. A significant difference in time in empathic contact and time in contact with the non-social object would suggest that the empathic contact is prosocial. The prosocial behavior most similar to empathic contact is exploratory sniffing, and a significant difference in empathic contact and exploratory sniffing would suggest that these are separate prosocial behaviors. If, as predicted, the empathic contact is a distinct prosocial behavior, without training or the ethological relevance of other prosocial behaviors, we conclude that it is a measure of empathy.

## Main text

### Methods

#### Husbandry

Study animals originated from an Urbana, IL wild stock and were used in a prior experiment [9]. The prairie vole is a rodent model for investigating prosocial behavior [22]. Prairie voles differ from traditional laboratory rodents in specific housing, diet and husbandry requirements [30–32].

#### Novel social test

Behavioral data recordings were taken for another, previously published experiment with different endpoints and goals [9]. Only the control animals of both sexes from that prior study were used for the work herein. Briefly, on post-natal day 30, the animals were tested for 1 h in a novel social arena as described previously [5, 33] and in Fig. 1, using a gently tethered, unrelated stimulus animal (same sex, size, and age matched). The tether was a restraint loosely fit around the stimulus animal’s neck on one end and fixed to the wall mounted non-social object (represented in Fig. 1 as an elongated grey oval with a red outline) preventing the test animal from entering the non-social chamber or the adjoining tube. Attached to the cage wall was a non-social object of the same material and luster as the tether that was not directly linked to the collar portion of the animal’s tether.

A treatment-blinded observer scored behaviors using Jwatcher v1.0 behavioral scoring software (UCLA) [34]. The measured behaviors included total test time spent in exploratory sniffing, chewing or pulling on tether/collar within 1.3 cm of the stimulus animal (empathic contact), and time spent engaging in contact with non-social object. An observer remained in the room for the duration of the test to monitor potential signs of aggression; no injury to any animals occurred.

#### Brain collection and preparation for immunohistochemistry

Brains were collected and processed as described in our prior publication [9]. Again, only the control animals were used for the experiments described herein. Briefly, the animals were euthanized between post-natal days 60–90. Each animal was given 0.05-mL buprenorphine ip and then deeply anesthetized 15 min later with 0.05 mL of ketamine–xylazine (at a concentration of 67.7 and 13.33 mg/kg, respectively) mixture administered sc. Brains were collected and immersion fixed for 24 h in 4% paraformaldehyde and transferred to fresh solution at 2 and 4 h. 30% buffered sucrose with 0.1% sodium azide was used to cryoprotect the brains, which were then shipped to the Patisaul Lab, where they were stored in fresh cryoprotectant overnight at 4 °C and then flash frozen and stored at − 80 °C. Brains were sectioned at 35 µm coronally on a frozen sliding microtome. Subsequently, sections for each individual brain corresponding to the regions of interest were collected and processed for immunohistochemistry.

#### Immunohistochemistry

For each test animal, 8 sequential sections of the PVN were collected and processed for immunohistochemical staining of AVP and OT using routine procedures described previously [9] using 1:12,000 monoclonal mouse anti-OT (catalog number MAB5296; Millipore) and 1:12,000 polyclonal rabbit anti-AVP (catalog number 20069; Immunostar). Sections were then washed and incubated for 120 min in a solution of Alexa Fluor 568 goat anti-mouse and Alexa Fluor 488 goat anti-rabbit secondary antibodies (both at 1:200). Immunohistochemical staining of TH was performed on two consecutive posterior PVN sections as described previously [9] with the polyclonal rabbit anti-TH (catalog number AB152; Millipore) primary antibody at 1:4000 and Alexa Fluor 488 donkey anti-rabbit secondary antibody at 1:200. These sections were also counterstained via a 45-s incubation with Hoechst (catalog number H3569; Invitrogen Life Technologies). After a final wash in cold KPBS, all PVN sections were mounted on Fisher super frost plus glass slides, coverslipped with a glycerol mountant, and stored at − 20 °C.

#### Quantification and analysis

The 8 sequential sections selected for OT immunoreactivity (OT-ir) and AVP immunoreactivity (AVP-ir) quantification encompassed the entire PVN, as previously described [9]. For the present study, the PVN subregional counts, established previously [9], were averaged within animal and then animal specific means were averaged by sex, so that each animal contributed one value to the final mean. The dopaminergic immunoreactive cell counts (TH-ir) are the same as previously reported [9].

### Statistical analysis

All analyses were done with IBM SPSS Statistic version 21. Novel social results were analyzed by a one-way ANOVA testing for significant (P ≤ 0.05) sex-specific differences (homogeneity of variance was validated using Levene’s test, there were no distribution effects of unequal sample sizes). Paired t tests (2-tailed) were performed within sex testing for significant (P ≤ 0.05) differences in total test time between empathic behavior and non-social object contact, or empathic behavior and exploratory sniffing (males n = 9, females n = 22).

Significant (P ≤ 0.05) sex-specific differences in numbers of OT-ir, AVP-ir and TH-ir neurons in the PVN were determined by a one-way ANOVA. Significant (P ≤ 0.05) within-sex differences in the number of neurons expressing OT, AVP and TH were determined by a one-way ANOVA (OT and AVP males and females n = 11, TH males and females n = 7).

### Results

**Fig. 2:**
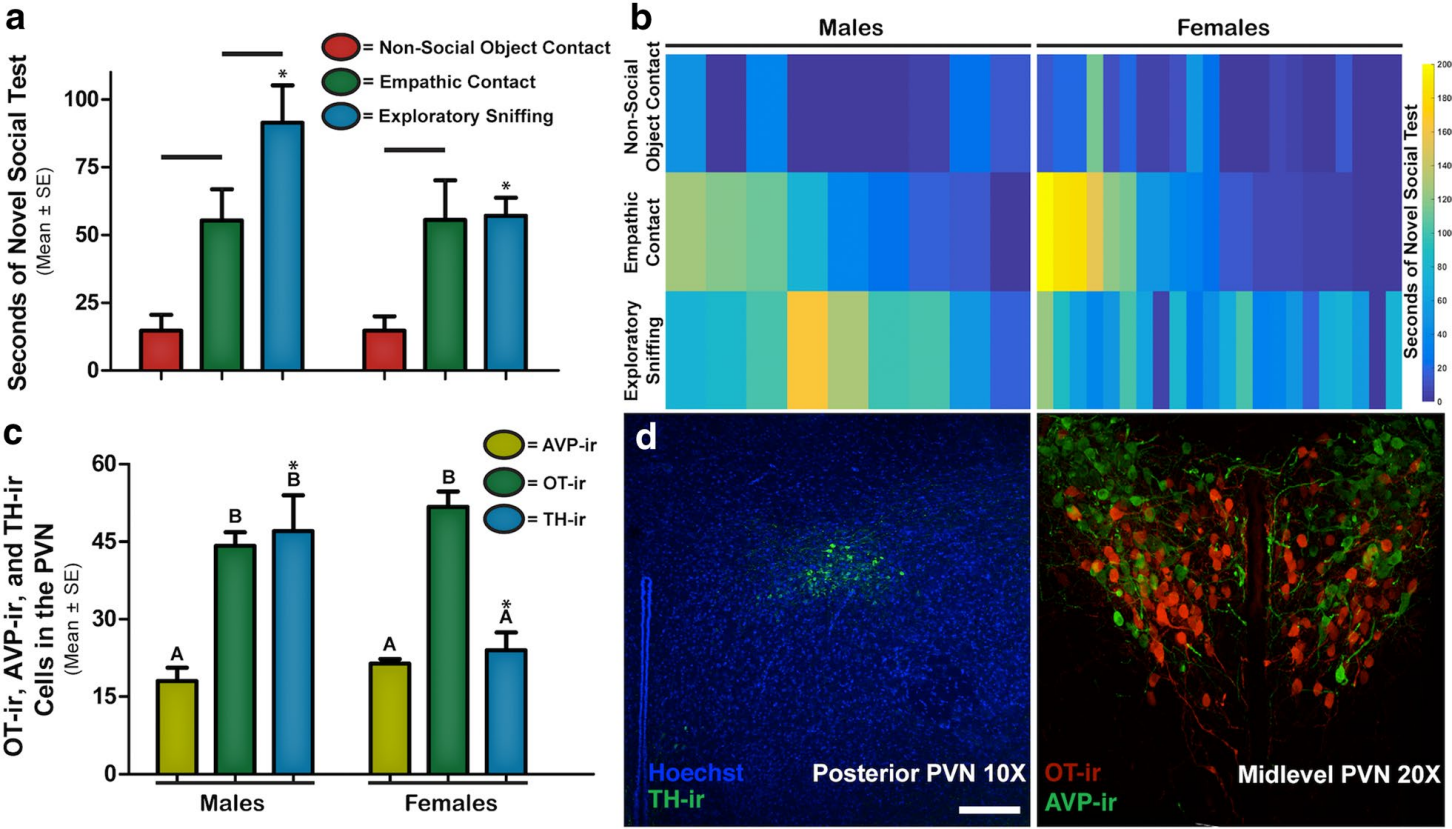
Results suggest that prairie voles will engage in unconditioned empathic contact and that number of OT and possibly AVP expressing neurons in the PVN is consistent with the pattern of empathic behavior, while the number of DA expressing neurons is consistent with overall exploratory behavior. **a** Seconds of Novel Social Test: “−” indicates significant paired difference, and “*” indicates significant difference between males and females (P < 0.05, males n = 9, females n = 22). **b** Two dimensional histogram of behavior durations, in seconds, for males (left) and females (right), in which each row is a separate behavioral measure, as indicated by the y-axis, and each column represents a single animal. **c** Neurons in PVN: Different letters indicate significant differences within sex, and “*” indicates significant difference between males and females (P < 0.05, OT and AVP males and females n = 11, TH males and females n = 7). **d** Posterior PVN TH-ir: Low magnification image represents of the posterior sub-region of the PVN in which TH-ir neurons were observed at low magnification scale bar 100 µm (left image). Medial PVN OT-ir and AVP-ir: Low magnification image represents the medial sub-region of the PVN in which OT-ir and AVP-ir neurons were observed at low magnification (right image) (confocal images were taken with the permission and generosity of Dr. Troy Ghashghaei at NCSU)

The behavioral results indicate that, both males (8 of 9) and females (20 of 22) engage in empathic contact by attempting to remove the tether from the stimulus animal (Fig. 2b). In the paired test results, males and females spent significantly more time engaging in contact with an object of potential empathic consequence versus a similar non-social object of no social consequence (paired t-test, male t_8_ = 3.77 P ≤ 0.005; female t_21_ = 3.09 P ≤ 0.05). However, only males spent significantly more time engaging in exploratory sniffing versus empathic contact (paired t-test, male t_8_ = − 2.29 P ≤ 0.05). Overall, males spent significantly more time engaging in exploratory sniffing than females (ANOVA F_(1,20)_ = 6.18, P ≤ 0.05) (Fig. 2a).

As expected, the immunohistochemistry results revealed that the number of OT-ir and AVP-ir neurons across the PVN are not sexually dimorphic, but that the density of dopaminergic neurons in the posterior PVN is sexually dimorphic (Fig. 2c). Significantly more TH-ir neurons were observed in males than females (ANOVA F(1,12) = 8.76 P ≤ 0.05). Males also had greater numbers of OT-ir neurons than AVP-ir neurons (paired t-test, male_OT-AVP_ t_10_ = 30 P ≤ 0.001). Additionally, there were more TH-ir neurons than AVP-ir neurons in males, but there was no significant difference between the number of OT-ir and TH-ir neurons. In the PVN of females, there were significantly fewer AVP-ir neurons and TH-ir neurons than OT-ir neurons (paired t-test, female_OT-AVP_ t_11_ = 12.23 P ≤ 0.001; female_OT-TH_ t_6_ = 4.17 P ≤ 0.05), with no significant difference between AVP-ir and TH-ir neurons. Taken together, the results of this study suggest sex-specific differences in the expression of prosocial behavior, with concordant patterns of sexually dimorphic significance in the underlying neurobiology of the PVN. It is important to note that while region and neuron specific histology provides a broad understand of the underlying mechanisms contributing to prosocial behavior, it is the OT and AVP signaling that guides behavior.

### Discussion

Here we used behaviorally observable exploratory sniffing to measure sexually dimorphic non-empathic prosocial behavior. Males spent significantly more time engaging in empathic contact than non-social object contact, suggesting that empathic contact is prosocial (Fig. 2a). Additionally, the significant difference between exploratory sniffing and empathic contact suggests that empathic contact is distinct from non-empathic prosocial behaviors (Fig. 2a).

Because empathy is regulated by OT and AVP, but not dopaminergic PVN neurons, in humans and voles [7, 12, 17–19] we hypothesized that if empathic contact is distinct from other prosocial behaviors, then the number of OT-ir, AVP-ir and TH-ir neurons in the PVN would support this difference. Our measure of non-empathic prosocial behavior, exploratory sniffing, was similar across sexes as were the number of TH-ir neurons in the PVN. However, as predicted, the number of TH-ir PVN neurons across sexes was not did not parallel the duration of empathic contact between males and females. This observation is consistent with prior work showing that dopamine expression in the cingulate cortex is linked to empathy, but expression in the PVN is not [21]. Our results support region-specific differences and suggest that dopaminergic neurons in the PVN may regulate exploratory sniffing and possibly other non-empathic prosocial behaviors.

Also consistent with our hypothesis, time spent in empathic contact and the number of AVP-ir and OT-ir neurons in the PVN, followed similar patterns across sexes (Fig. 2c). These data support prior work showing that empathy is regulated by oxytocinergic and vasopressinergic systems in the PVN of humans, rats and prairie voles [7, 8, 18, 19]. The number of PVN neurons expressing AVP and OT is independent of sex and consistent with the time spent in empathic contact versus contact with a non-social object across sexes. Finally, the neuroanatomical mechanisms that regulate empathy in prairie voles [7], and the time spent in empathic contact, support the prediction that empathic contact is a distinct prosocial behavior, independent of training or known ethological relevance, and is a measure of empathy. All supporting data can be accessed through the Additional file 1.

## Limitations

The cell counts used in this study provide strong correlative evidence for the role of PVN OT, AVP, and TH neurons in empathic behavior, but to demonstrate causal influence of these neuronal populations would require in vivo manipulations of the discussed cell populations. Reduced empathic contact concurrent with optogenetic silencing of PVN OT or AVP neurons using a noninvasive red-shifted microbial rhodopsin [35] would provide strong evidence for a causal role of these cells in empathy. To a lesser extent the use of pharmacological manipulations, such as OT or AVP antagonists could provide similar support, but could not account for off target effects from the peripheral nervous system, or other brain regions that express OT such as the olfactory tubercle [36].

## Additional file

**Additional file 1.** Neuroanatomy: The number of cells counted for OT, AVP, and TH in the PVN. Behavior: the total durations of time spent engaging in specific behaviors compared within the text.

## Abbreviations

OT: oxytocin
PVN: paraventricular nucleus of the hypothalamus
AVP: arginine vasopressin
TH: tyrosine hydroxylase
-ir: immunoreactive
